# Creativity in the Wild: Improving Creative Reasoning through Immersion in Natural Settings

**DOI:** 10.1371/journal.pone.0051474

**Published:** 2012-12-12

**Authors:** Ruth Ann Atchley, David L. Strayer, Paul Atchley

**Affiliations:** 1 Department of Psychology, University of Kansas, Lawrence, Kansas, United States of America; 2 Department of Psychology, University of Utah, Salt Lake City, Utah, United States of America; Goldsmiths, University of London, United Kingdom

## Abstract

Adults and children are spending more time interacting with media and technology and less time participating in activities in nature. This life-style change clearly has ramifications for our physical well-being, but what impact does this change have on cognition? Higher order cognitive functions including selective attention, problem solving, inhibition, and multi-tasking are all heavily utilized in our modern technology-rich society. Attention Restoration Theory (ART) suggests that exposure to nature can restore prefrontal cortex-mediated executive processes such as these. Consistent with ART, research indicates that exposure to natural settings seems to replenish some, lower-level modules of the executive attentional system. However, the impact of nature on higher-level tasks such as creative problem solving has not been explored. Here we show that four days of immersion in nature, and the corresponding disconnection from multi-media and technology, increases performance on a creativity, problem-solving task by a full 50% in a group of naive hikers. Our results demonstrate that there is a cognitive advantage to be realized if we spend time immersed in a natural setting. We anticipate that this advantage comes from an increase in exposure to natural stimuli that are both emotionally positive and low-arousing and a corresponding decrease in exposure to attention demanding technology, which regularly requires that we attend to sudden events, switch amongst tasks, maintain task goals, and inhibit irrelevant actions or cognitions. A limitation of the current research is the inability to determine if the effects are due to an increased exposure to nature, a decreased exposure to technology, or to other factors associated with spending three days immersed in nature.

## Introduction

Our environment plays a critical role in how we think and behave. The modern environment experienced by most individuals living in urban or suburban settings can be characterized by a dramatic decrease in our exposure to natural settings and a correlated increase in exposure to a technology intense environment. Data suggest that children today spend only 15–25 minutes a day in outdoor play and sports [1] and this number continues to decline. There has been a 20% decline in per capita visits to national parks since 1988, and a 18–25% decline in nature-based recreation since 1981 [2]. Concurrently, eighty percent of kindergarten aged children are computer users (USDE, 2005) and the average 8–18 year old now spends over seven and a half hours per day using one or more types of media (TV, cell phones, computers) [3], while adults likely spend more time engaged with different forms of media technology (for example see OFCOM Communications Market Report) [4].

Attention Restoration Theory (ART) [5] suggests that nature has specific restorative effects on the prefrontal cortex-mediated executive attentional system, which can become depleted with overuse. High levels of engagement with technology and multitasking place demands on executive attention to switch amongst tasks, maintain task goals, and inhibit irrelevant actions or cognitions. ART suggests that interactions with nature are particularly effective in replenishing depleted attentional resources. Our modern society is filled with sudden events (sirens, horns, ringing phones, alarms, television, etc.) that hijack attention. By contrast, natural environments are associated with a gentle, soft fascination, allowing the executive attentional system to replenish. In fact, early studies have found that interacting with nature (e.g., a wilderness hike) led to improvements in proof reading [6], control of Necker Cube pattern reversals [7], [8], and performance on the backwards digit span task [9]. Laboratory-based studies have also reported that viewing slides of nature improved sustained attention [10] and the suppression of distracting information [9]. However, the impact of more sustained exposure to natural environments on higher-level cognitive function such as creative problem solving has not been explored.

To empirically test the intriguing hypotheses that complex cognition is facilitated by prolonged exposure to natural settings and the parallel release from technology immersion, the current research utilized a simple and ecologically valid paradigm of measuring higher order cognitive production in a pre-post design looking at the cognitive facilitative effects of immersion in nature. To the best of our knowledge, this is the first attempt to examine changes in higher-order cognitive production after sustained exposure to nature, while participants are still in the natural environment. The higher order cognitive task used was the Remote Associates Test (RAT) developed by Mednick [11], [12], which has been widely used as a measure of creative thinking and insight problem-solving. Utilizing insight, problem solving, and convergent creative reasoning to effectively connect the cues provided through a mediated relationship (for example: SAME/TENNIS/HEAD = MATCH) is thought to draw on the same pre-frontal cortical structures that are hypothesized to be overtaxed by the constant demands on our selective attention and threat detection systems from our modern, technology-intensive environment.

## Methods

Fifty-six (26 Female, average age = 28 years) adults involved in wilderness expeditions run by Outward Bound (http://www.outwardbound.org/) participated in the study. Informed voluntary consent was provided in writing by the Outward Bound organization and was obtained for all participants in the study. The study utilized a between subjects design with 8 hiking groups (half randomly assigned to the pre-hike group and half to the in-hike group). The pre-hike groups backpacked in Alaska (n = 8), Colorado (n = 10), or Maine (n = 6) and the in-hike groups backpacked in Alaska (n = 9), Colorado (n = 14) or Washington (n = 9) and there was no communication between hiking groups. All hikes involved backpacking in the wilderness for 4–6 days and all participants were prohibited from using any electronic technology during the outing. A between-subjects design was selected to avoid unwanted carry-over effects (including collaboration between participants).

The pre-hike participant sample was composed of twenty-four participants (11 Female, average age = 34) and the in-hike group was made up of 32 participants (15 Female, average age = 24). Because age has an effect on the task, age was run as a covariate in subsequent analyses. The pre-hike group completed the RAT measure on the morning before they began their backpacking trip. The in-hike group completed the RAT measure in the morning of the fourth day or their trip. All participants were given an unlimited amount of time to complete 10 Remote Associate Items [13] and the primary dependent variable was the number of correct items provided out of 10 possible. All RAT tasks were completed independently and both analysis of the responses provided and Outward Bound councilors indicated that no collaboration happened between participants.

## Results

A simple between-participant ANOVA was utilized. As anticipated, age of participant did significantly influence hit rate for the RAT measure (*F*(1,53) = 7.20, *p*<.01, *MS* = 32.88) and therefore was included as a covariate in the analysis of Group effects. In this analysis we found that the pre-hike group were able to answer fewer RAT items (*M* = 4.14, *SD* = .46) than the in-hike group (*M* = 6.08, *SD* = .39), *F*(1,53) = 9.71, *p*<.01, *MS* = 44.33, Cohen’s D = 0.86. This represents a 50% increase in performance after four days of exposure to nature.

## Discussion

Testing higher-order cognitive skills in a natural environment is a challenge. The current study is unique in that participants were exposed to nature over a sustained period and they were still in that natural setting during testing. Despite the challenging testing environment, the current research indicates that there is a real, measurable cognitive advantage to be realized if we spend time truly immersed in a natural setting. Further, unlike previous research in which cognitive changes were measured with laboratory tests of attentional function and/or laboratory surrogates for exposure to nature, the current work demonstrates that higher-order cognitive skills improve with sustained exposure to a natural environment. The current study lays the groundwork for further work examining the mechanism of this effect by providing evidence and a method by which improved cognitive performance can be examined in the wild.

There are multiple candidates for potential mechanisms underlying the effects observed here and in other studies. It is likely that the cognitive benefits of nature are due to a range of these mechanisms and it will require a sustained program of research to fully understand this phenomenon. One suggestion is that natural environments, like the environment that we evolved in, are associated with exposure to stimuli that elicit a kind of gentle, soft fascination, and are both emotionally positive and low-arousing [9]. It is also worth noting that with exposure to nature in decline, there is a reciprocal increase in the adoption of, use, and dependency upon technology [14]. Thus, the effects observed here could represent either removal of the costs associated with over-connection or a benefit associated with a return to a more positive/low-arousing restorative environment.

Exposure to nature may also engage what has been termed the “default mode” networks of the brain, which an emerging literature suggests may be important for peak psychosocial health [15]. The default mode network is a set of brain areas that are active during restful introspection and that have been implicated in efficient performance on tasks requiring frontal lobe function such as the divergent thinking task used here [16]. On a hike or during exposure to natural stimuli which produce soft-fascination, the mind may be more able to enter a state of introspection and mind wandering which can engage the default mode. Interestingly, engaging the default mode has been shown to be disrupted by multimedia use, which requires an external attentional focus, again pointing to the possibility that natural environments such as those experienced by the current participants may have both removed a cost (technology) and added a benefit (activation of brain systems that aid divergent thinking).

This study is the first to document systematic changes in higher-level cognitive function associated with immersion in nature. There is clearly much more research to be done in this area, but the current work shows that effects are measurable, even in completely disconnected natural environments, laying the groundwork for further studies. Much about our cognitive and social experience has changed in our current technology-rich society and it is challenging to fully assess the health costs associated with these changes. Nevertheless, the current research establishes that there are cognitive costs associated with constant exposure to a technology-rich, suburban or urban environment, as contrasted with exposure to the natural environment that we experience when we are immersed in nature. When our research participants spent four days in a natural setting, absent all the tools of technology, the surrounding natural setting allowed them to bring a wide range of cognitive resources to bear when asked to engage in a task that requires creativity and complex convergent problem solving.

A limitation to the current research is the inability to determine if the effects are due to an increased exposure to nature, to a decreased exposure to technology, or to other factors associated with spending three days immersed in nature. In the majority of real-world multi-day hiking experiences, the exposure to nature and technology are inversely related and we cannot determine if one factor has more influence than another. From a scientific perspective, it may prove theoretically important to understand the unique influences of nature and technology on creative problem solving; however, from a pragmatic perspective these two factors are often so strongly interrelated that they may be considered to be different sides of the same coin. We suggest that attempts to meaningfully dissociate the highly correlated real-world effects of nature and technology may be like asking Gestalt psychologists whether figure or ground is more important in perceptual grouping.

In principle, a 2×2 factorial study with high or low levels of nature (N+ or N−, respectively) and high or low levels of technology (T+ or T−, respectively) could shed light on the issue of dissociating the effects of nature and technology on complex problem solving. In the majority of real-world urban environments, T+N− is the norm whereas T−N+ is more common in the outdoor settings. Our research demonstrates that interacting for three days in T−N+ environments (i.e., the in-hike group) results in significant improvements in creative problem solving compared to T+N− environments (i.e., the pre-hike group). The T+N+ condition reflects an interesting situation where the interloper brings technology with them on the hike (assuming there is service and power) and, based on ART, we predict that interacting in this sort of environment would not benefit creative problem solving. The T−N− condition reflects a different scenario in which people interact in urban settings without the use of technology – a condition that is becoming increasingly rare in the modern world. Based upon ART, which places an emphasis on natural environments for maximal restoration, we predict that T−N+ condition would result in superior creative problem solving compared to T−N− condition (assuming that we could convince people to part with their digital technology for three full days). Future research will be required to evaluate these latter predictions.
